# Dataset for the simulated biomass pyrolysis in rotary kilns with varying particle residence time distributions

**DOI:** 10.1016/j.dib.2021.107603

**Published:** 2021-11-23

**Authors:** Mario Pichler, Bahram Haddadi, Christian Jordan, Hamidreza Norouzi, Michael Harasek

**Affiliations:** a Institute of Chemical, Environmental and Bioscience Engineering, TU Wien, Getreidemarkt 9/166, Vienna 1060, Austria; b Center of Engineering and Multiscale Modeling of Fluid Flow (CEMF), Department of Chemical Engineering, Amirkabir University of Technology (Tehran Polytechnique), PO Box: 15875-4413, Hafez 424, Tehran, Iran

**Keywords:** Residence time distribution, Biomass, Pyrolysis, Rotary kiln, Numerical simulation

## Abstract

Slow pyrolysis of biomass is commonly performed in rotary kilns. The effect of the particle residence time distribution on biomass conversion is often neglected when numerically modeling such systems. But this effect might be significant under certain conditions.

The data presented here are results of numerical simulation of the biomass pyrolysis in rotary kilns under numerous operating conditions and levels of axial dispersion of biomass particles. The varied operating conditions are the kiln diameter (D=0.1–1 m), the ratio of particle to kiln diameter ($\text{d}/\text{D}=5\;\times \;10^{-3}$–$40\;\times \;10^{-3}$), the ratio of kiln length to kiln diameter (L/D=1–10), the kiln’s inclination angle (β=0.1–8${}^{\circ }$), the Froude number ($\text{Fr}=10^{-3}$–$10^{-2}$), the rotational Reynolds number ($\text{Re}=10^{2}$–$16\;\times \;10^{3}$), and the Péclet number (Pe=5–100). Data of 13,851 single case simulations are provided with this article. This includes the mean particle residence time, gas, bed and kiln wall temperatures, solid and gaseous species mass flows, heat fluxes, and the solid bed height over the kiln length.

These comprehensive data have the potential to help in modeling, design, analysis, and optimization of rotary kilns used for the pyrolysis of biomass.

The main characterization and interpretation is presented in the related main research paper by Pichler et al. (2021)[1].

## Specifications Table

**Table:** 

Subject	Engineering
Specific subject area	Slow pyrolysis of biomass in rotary kilns under varying dimensionless conditions.
Type of data	Table Graph
How data were acquired	One dimensional numerical steady state simulation of biomass pyrolysis in externally heated rotary kilns. Software: Python, *scipy*.
Data format	Raw
Parameters for data collection	Kiln diameter D: 0.1, 0.55, and 1 m Ratio of particle to kiln diameter d/D: 5 $\times 10^{-3}$, 22.5 $\times 10^{-3}$, and 40 $\times 10^{-3}$ Ratio of kiln length to kiln diameter L/D: 1, 5.5, and 10 Kiln inclination angle β: 0.1, 4.05, and 8${}^{\circ }$ Froude number Fr: $10^{-3}$, 5.5 $\times 10^{-3}$, and $10^{-2}$ Rotational Reynolds number $Re_{\omega }$: 100, 8050, and 16,000 Péclet number Pe: 5–100
Description of data collection	For each of the possible 729 combinations of the six parameters (D, d/D, L/D, β, Fr, Re${}_{\omega }$), 19 numerical simulations with varying Péclet numbers were performed. Species mass fluxes, heat fluxes, temperatures, and the mean particle residence time over the kiln length were determined from numerical simulation.
Data source location	Institution: Institute of Chemical, Environmental & Bioscience Engineering, TU Wien City/Town/Region: Vienna Country: Austria
Data accessibility	Data and Code: With the article (supplementary material) and on public repositories. Data [4]: Repository name: Mendeley Data Data identification number: https://doi.org/10.17632/j3zm2xv53y.1 Direct URL to data: https://doi.org/10.17632/j3zm2xv53y.1 Code [5]: Repository name: GITHUB Data identification number: https://doi.org/10.5281/zenodo.5638119 Direct URL to data: https://doi.org/10.5281/zenodo.5638119
Related research article	Mario Pichler, Bahram Haddadi, Christian Jordan, Hamidreza Norouzi, Michael Harasek “Influence of particle residence time distribution on the biomass pyrolysis in a rotary kiln” Journal of Analytical and Applied Pyrolysis 2021, 158, 105171 DOI: https://doi.org/10.1016/j.jaap.2021.105171 Ref.: Pichler et al. [1]

## Value of the Data

• The data provides particle mean residence time, biomass conversion, biomass and gas temperature, and heat flux data of the biomass pyrolysis in rotary kilns including, commonly neglected effects of the particle residence time distribution. Data is provided for 729 cases with numerous operating conditions.
• The data will appeal to chemical engineers, process engineers, and researchers involved in the modeling and design of rotary kilns.
• The data can be used to decide under which conditions the particle residence time distribution should be considered when modeling the process. It also helps to design rotary kilns concerning the lifter design, which significantly influences the particle residence time distribution.
• The provided data helps to analyze, model, and optimize the biomass pyrolysis process in rotary kilns.

## 1 Data Description

The data set consists of

1. 729 case directories (0–728),
2. a directory called ‘bedHeights’,
3. and a list of all provided simulation cases (‘listOfCases.csv’).

*ad 1* Each case directory contains a file where the dimensioned case settings are provided and a sub-directory containing simulation results for 19 Péclet numbers (‘log_Pe5’–‘log_Pe100’). The dimensioned case settings include the dynamic angle of repose of the particles *phi* in ${}^{\circ }$, the kiln inclination angle *beta* in ${}^{\circ }$, the feed flow *Feed* in kg/s, the kiln diameter *D* in m, the kiln length *L* in m, the particle diameter *d_p*, in m, and the rotational speed of the kiln *n* in 1/s.

**Fig. 1:**
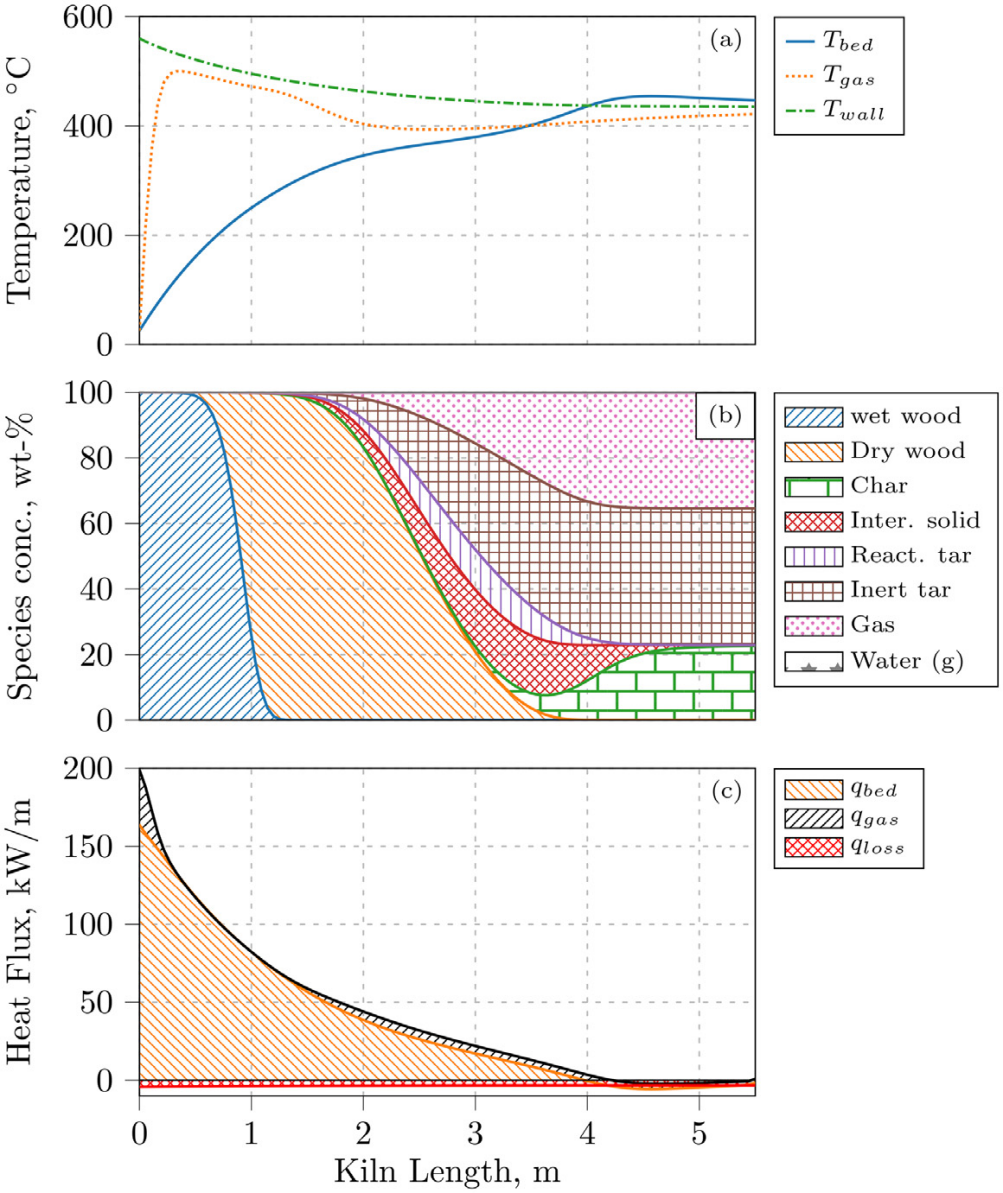
Simulation results along the kiln for case 493 with Pe = 100. (a) Temperatures of solid, gas, and wall. (b) Solid and gas phase species concentrations. (c) Heat flux to bed q${}_{bed}$, gas phase q${}_{gas}$, and surroundings q${}_{loss}$. q${}_{gas}$ is stacked on top of q${}_{bed}$ for better visualization.

Simulation results, provided in ‘log_Pe5’–‘log_Pe100’, include the mean residence time *Tau* in s, the species mass flows *F_i* in kg/s, the bed, gas, and wall temperatures *T_b, T_g*, and *T_w* in K, and the heat fluxes *q_bed, q_gas*, and *q_loss* in kW/m over the kiln length. Data was sampled at up to 900 discrete positions along the kiln. As an example, data for case 493 (Pe = 100) is plotted in Fig. 1.

Some combination of simulation settings led to overfilling of the kiln with particles or emptying of the kiln. In such cases, simulation led to no results, thus no results files, but only the dimensioned simulation conditions are provided.

**Fig. 2:**
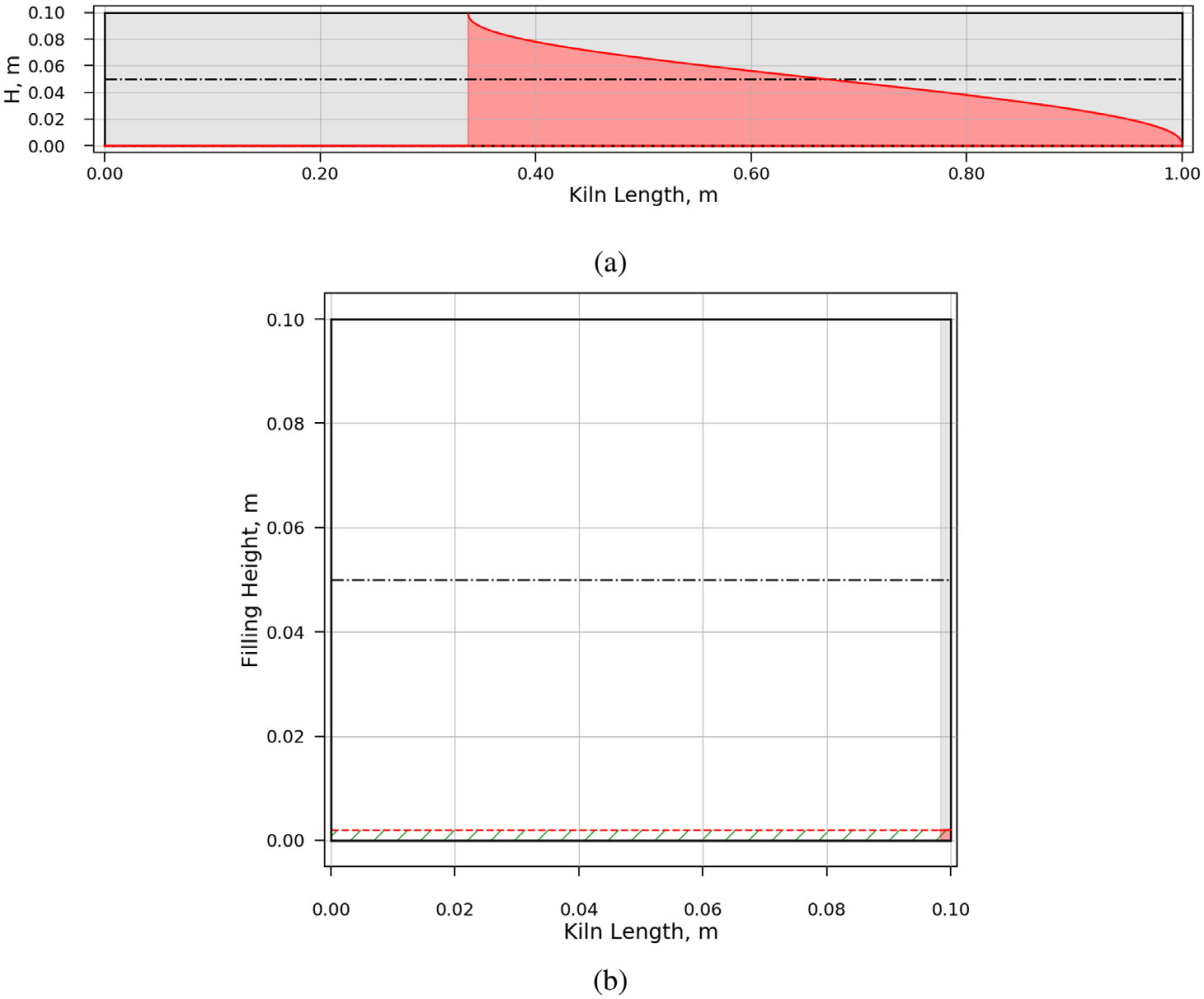
Simulated bed height over kiln length. (a) Case 2. Overfilling is indicated by the bed height colored in red. (b) Case 93. Emptying of the kiln is indicated by the green hatched area. (For interpretation of the references to color in this figure legend, the reader is referred to the web version of this article.)

*ad 2* In the ‘bedHeights’ directory, plots of the bed height over the kiln length are provided. The bed height is a function of operating condition and not influence by the Péclet number. Thus only one plot for each of the case 729 is provided. For cases that led to overfilling of the kiln, the bed height is colored red (e.g., case 2, Fig. 2(a)). Emptying of the kiln is indicated by the green hatched area (e.g., case 93, Fig. 2(b)). The red dashed line represents the dam height.

*ad 3* The file ‘listOfCases.csv’ contains the case ID and the varied operating conditions of all simulated cases. Operating conditions are the kiln diameter D, the ratio of particle to kiln diameter d/D, the ratio of kiln length to kiln diameter L/D, the kiln inclination angle β, the Froude number Fr, and the rotational Reynolds number $Re_{\omega }$.

## 2 Experimental Design, Materials and Methods

**Fig. 3:**
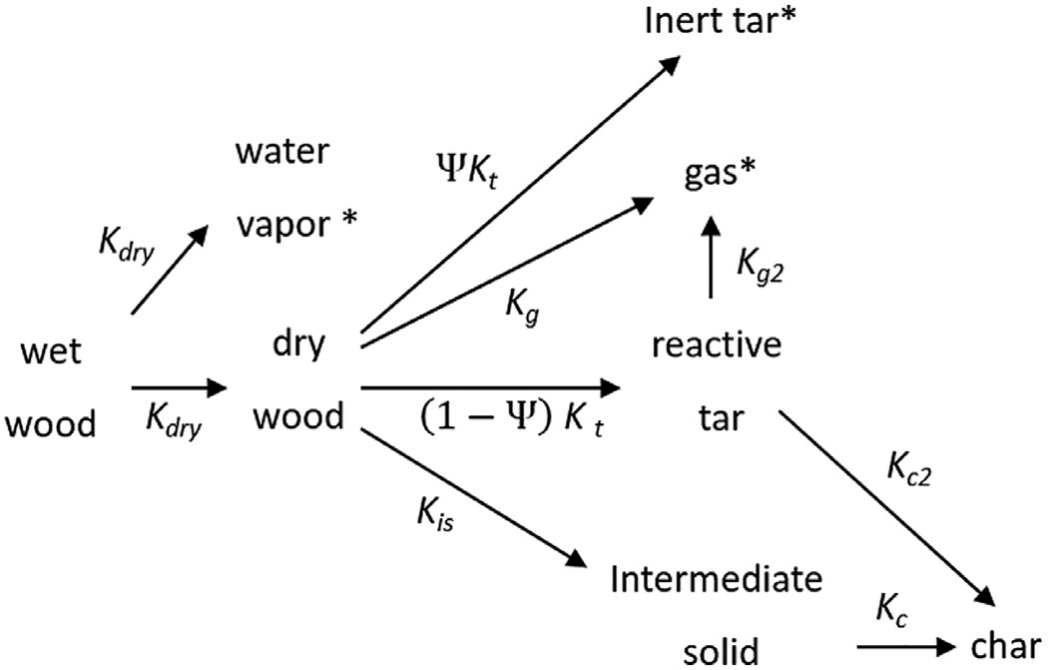
Reaction scheme based on Babler et al. [2] and Sousa and Azevedo [3]. * gaseous species. Adapted from Pichler et al. [1].

Data was gathered from one dimensional numerical steady state simulations of a rotary kiln. The model considers a rotary kiln of the length L and the diameter D, rotating with an angular velocity ω. A volumetric stream of particles Q enters at one side of the kiln and is discharged via a dam at the other side. A small stream of nitrogen is fed to the kiln alongside the particles. A hot flue gas stream is used to heat the kiln in co-current flow. Heat transfer to the kiln wall is assumed to be fast, and the wall heat capacity is neglected. Uniform temperatures of the solid bed phase domain T${}_{b}$ and of the gas phase domain T${}_{g}$ are assumed at each position along the kiln, indicating intense mixing in the radial direction. The residence time distribution of the particles is modeled using the axial dispersion model. Biomass drying and conversion are modeled using the reaction scheme shown in Fig. 3. Reactions are assumed to take place solely in the bed domain. A detailed description of the model, the boundary conditions, the model parameters, and the solution strategy are presented by Pichler et al. [1].

Simulations were carried out with conditions, models, and thermo-physical properties described in Pichler et al. [1]. Operating conditions were changed based on parameters shown in Table 1. For each of the 729 possible combinations of parameters, the axial dispersion of the solid particles was varied using 19 different Péclet numbers, resulting in a total of 13,851 single case simulations.

**Table 1:** Parameter values used in the study. Adapted from Pichler et al. [1].

Parameter	Low	Medium	High
D, m	0.1	0.55	1
d/D, -	$5\times 10^{-3}$	$22.5\times 10^{-3}$	$40\times 10^{-3}$
L/D, -	1	5.5	10
β, -	0.1	4.05	8
Fr, -	$10^{-3}$	$5.5\times 10^{-3}$	$10^{-2}$
$Re_{\omega }$	100	8050	16,000
Pe, -	5–100		

As described earlier, some combinations of settings led to overfilling or emptying of the kiln. The kiln was considered as overfilled when the local bed height exceeds 0.9 times the kiln diameter. The kiln was considered empty if the local bed height was smaller than 0.95 times the dam height. In these cases, no simulation results could be obtained.

## Ethics Statement

Not applicable.

## CRediT authorship contribution statement

**Mario Pichler:** Conceptualization, Methodology, Software, Formal analysis, Writing – original draft. **Bahram Haddadi:** Conceptualization, Methodology, Software, Writing – review & editing. **Christian Jordan:** Writing – review & editing, Project administration. **Hamidreza Norouzi:** Data curation, Writing – review & editing. **Michael Harasek:** Resources, Supervision, Funding acquisition.

## Declaration of Competing Interest

The authors declare that they have no known competing financial interests or personal relationships which have, or could be perceived to have, influenced the work reported in this article.

## Appendix A Supplementary materials

Supplementary Data S1 Supplementary Raw Research Data. This is open data under the CC BY license http://creativecommons.org/licenses/by/4.0/ Supplementary Data S1

## References

[1] Pichler M., Haddadi B., Jordan C., Norouzi H., Harasek M. (2021). Influence of particle residence time distribution on the biomass pyrolysis in a rotary kiln. J. Anal. Appl. Pyrolysis.

[2] Babler M.U., Phounglamcheik A., Amovic M., Ljunggren R., Engvall K. (2017). Modeling and pilot plant runs of slow biomass pyrolysis in a rotary kiln. Appl. Energy.

[3] Sousa N., Azevedo J.L. (2016). Model simplifications on biomass particle combustion. Fuel.

[4] Pichler M., Haddadi B., Jordan C., Norouzi H., Harasek M. (2021). Dataset for the simulated biomass pyrolysis in rotary kilns with varying particle residence time distributions. Mendeley Data.

[5] Mptutvt, mptutvt/rotarypyrolysis: First code release (v1.0.0), Zenodo (2021), doi:10.5281/ZENODO.5638119.

